# Predictors of COVID-19 vaccine uptake among people who use substances: a case study in Tehran

**DOI:** 10.1186/s13011-024-00596-9

**Published:** 2024-02-26

**Authors:** Salah Eddin Karimi, Sina Amadi, Zahra Rampisheh, Batool Tayefi, Neda Soleimanvandiazar, Peter Higgs, Arash Tehrani-Banihashemi, Ahmad Hajebi, Marzieh Nojomi, Gelavizh Karimijavan

**Affiliations:** 1 Social Determinants of Health Research Center, Health Management and Safety Promotion Research Institute, Tabriz University of Medical Sciences, Tabriz, Iran; 2 Social Development and Health Promotion Research Center, Health Institute, Kermanshah University of Medical Sciences, Kermanshah, Iran; 3 Preventive Medicine and Public Health Research Center, Psychosocial Health Research Institute, Department of Community and Family Medicine, School of Medicine, Iran University of Medical Sciences, Tehran, Iran; 4 Preventive Medicine and Public Health Research Center, Psychosocial Health Research Institute, Department of Community and Family Medicine, School of Medicine, Iran University of Medical Sciences, P.O Box: 14665-354, Tehran, 1449614535 Iran; 5 Department of Public Health, La Trobe University, Victoria, Australia; 6 Behaviours and Health Risks Program, Burnet Institute, 85 Commercial Road, Melbourne, VIC 3004 Australia; 7 Department of Public Health, La Trobe University, Plenty Rd & Kingsbury Dr, Bundoora, Melbourne, VIC 3086 Australia; 8 Research Center for Addiction and Risky Behaviors (ReCARB), Psychosocial Health Research Institute, Department of Psychiatry, School of Medicine, Iran University of Medical Sciences, Tehran, Iran; 9 Department of Sociology and Anthropology, Nipissing University, North Bay, ON Canada; 10 Department of Speech Therapy, Rehabilitation Faculty, Tabriz University of Medical Sciences, Tabriz, Iran

**Keywords:** Vaccination, Covid-19, Substance use, People Who Use Substances, People Who Inject Drugs

## Abstract

**Background:**

Vaccination is one of the most effective ways to manage infectious disease epidemics such as Covid-19. However, the low rates of vaccination in populations at risk including people using illicit substances, hinders the effectiveness of preventive vaccines in reducing transmission. The aim of this study was to investigate the rate of Covid-19 vaccination and its related factors among people who use substances in Tehran, Iran.

**Methods:**

Between July and December 2022, 386 people who use substances aged ≥ 18 years old were recruited by convenience street-based sampling in Tehran. The outcome variable in this study was self-reported completion of at least two doses of the Covid-19 vaccine. Logistic regression was used to investigate the factors related to Covid-19 vaccination. Data were analyzed using SPSS software version 20 at the 0.05 level of significance. As a measure of risk, 95% Confidence interval (CI) was used. The level of significance was considered at 0.05.

**Results:**

Almost three-quarters (*n* = 286) of the participants reported receiving at least two doses of the Covid-19 vaccine (95% CI, 70.2–79.3). Those participants with high school diplomas were 1.17 times more likely than less educated participants to report having had 2 vaccinations (OR of 1.17, CI 95%: 1.03–1.81). Participants with a higher mean score of having a positive attitude towards Covid-19 vaccination were more likely to have received a vaccination (OR of 1.12, CI 95%: 1.08–1.17). Ethnicity was also an influential variable, people with non-Fars ethnicity were less likely to be vaccinated than those of Fars ethnicity (OR of 0.33, CI 95%: 0.13–0.81). People with higher-than-average monthly income were more likely to report vaccination than those with low monthly incomes (OR of 1.27, CI 95%: 1.09–1.8). Also, participants reporting less access to vaccination centers had a lower chance of reporting having been vaccinated than those who reported high access to vaccination centers (OR of .17, CI 95%: .08-.36).

**Conclusions:**

Covid-19 vaccine uptake was found to be relatively high among people using illicit substances in this study. Higher levels of education, Fars ethnicity, higher income levels, having a positive attitude towards vaccination and access to vaccination centers were the most important predictors of Covid-19 vaccination in this study.

## Background

The Covid-19 pandemic is a modern global crisis endangering the life, health and well-being with particularly negative effects on the lives of people living with chronic health issues including people who use illicit substances [1]. Previous research has shown that the Covid-19 pandemic led to the increased use of alcohol and other substances in a range of settings globally [2–5]. It has also been well established that substance use has negative effects on health with devastating social harms [6–10]. So, effective, practical, and sustainable policies and practices is necessary to prevent substance use and all the problems and risk factors that increase substance use and its consequences [11, 12]. Preventive vaccination is a vital strategy in managing the challenges of Covid-19 among people who use substances. Vaccination is well known to be an effective tool in the control of Covid-19 transmission, hospitalization and mortality [13]. Previous research has highlighted low vaccination rates are one of the major factors contributing to the transmission of Covid-19 [14].

Increasing the uptake of the Covid-19 vaccination is one way to slow the spread of associated illness and to reduce the most negative consequences of infection. The acceptance and uptake of some vaccines such as influenza have been found to be less than optimal in different population groups, with vulnerable populations showing high rates of mistrust [15]. Concerns about non-compliance with prevention and treatment for people who use illicit substances have been noted amongst health care workers [16]. There are a range of factors affecting vaccine uptake. These include age, race, physicians’ profession, seasonal influenza vaccine compliance, Covid-19 patient care involvement, confidence in Covid-19 vaccine safety and effectiveness, previous Covid‑19 infection, negative economic impacts of the pandemic, reporting a negative opinion about Covid‑19 vaccine safety, trust in Covid‑19 vaccine information when delivered by the doctor, trust in the government’s actions, having health insurance, knowing more vaccinated people and having a history of imprisonment that have been found to be associated with Covid-19 vaccine compliance in the previous studies [4, 17–22]. Available literature has shown that, in general, people using illicit substances have lower hepatitis B and influenza vaccination rates than the general population [23, 24]. People who use illicit substances are among the most marginalized social groups and may live with compromised immune systems and hence have greater vulnerability to pathogens [25]. They often have extensive experiences of homelessness, due to living in shelters or crowded centers and other poor social conditions. They may also live with chronic and communicable diseases and together with their living circumstances these increase the opportunities for Covid-19 exposure and infection [20, 26]. Combine this with lower health literacy than the general population and higher levels of mistrust of health services, experiences of stigma, and limited access to mass media and information, all impact the acceptance of vaccines across this population [25, 27].

Population estimates for Iran suggest that there are between 1.2 and 2 million people using illicit drugs [28, 29], about 20–25% of whom are thought to inject [28, 30–34]. As vaccine acceptance in Iran's general population is still not at the optimal level, and because vaccination uptake for people using illicit substances should be a priority of the health system [35, 36], it is important to understand rates of Covid-19 vaccination and its related factors for people using substances in Iran. Thus, the aim of the current study was to investigate the rate of Covid-19 vaccination uptake and related factors among a convenience sample of people using substances.

## Methods

### Sampling and participants

In this cross-sectional study, street outreach interviews with people who use substances aged over 18 years old took place between July and December 2022. Using street-based sampling potential participants were approached in various locations, such as streets, parks, shelters, motels, river canyons, and vacant lands and asked about their interest in study participation. Data were on all weekdays, in the morning and evening. We used street-based sampling, as responses to sensitive topics are more reliable than phone or home-based survey recruitment for people using substances in Iran [37].

According to the below formula for estimating the sample size, based on the vaccination prevalence (*P* = 57%) in Iran in March 2021, the sample size with a confidence interval of 95% (Z = 1.96) and an error level of less than 0.05 (D = 0.05) was calculated as 380. As the attrition was considered 10%, the finalized sample size was estimated at 420 people.

$$n=\frac{{z}_{1-\frac{a}{2}}^{2} p(1-p)}{{d}^{2}}$$

### Inclusion and exclusion criteria

Participants were eligible if they reported using illicit substances in the past month (at least weekly for the month prior to answering the questionnaire) and must have been living in Tehran for at least a year, and be aged over 18 years. In this study, any type of substances whose consumption is illegal in Iran were considered illicit substances. Substances included the following; (a) opium (locally known as “Teriak,” which has two forms: “Sookhteh” and Shire); (b) heroin and/or crack (H-C); (c) stimulants including methamphetamine, ice, ecstasy (X pill), cocaine or coke, Ritalin or methylphenidate; (e) hallucinogens including lysergic acid diethylamide (LSD), ketamine, and hallucinogenic fungi (ie, mushrooms); and (f) alcohol [37].

Exclusion criteria included unwillingness to participate in the study, lack of verbal communication ability, and lack of cognitive ability to answer survey questions.

Data were collected from the participants through face-to-face interviews after obtaining verbal informed consent. A small gift was provided to the participants as reimbursement for their time and expertise. The study was approved by the Human Research Ethics Committee, from the Iran University of Medical Sciences (IR.IUMS.REC.1401.447).

### Variables and measures

The outcome variable of this study was Covid-19 vaccination, which was measured by self-report completion of Covid19 vaccine (at least two doses) using the following question: “Have you received 2 or more doses of the Covid-19 vaccine? Yes/No”.

For independent variables data collection, a checklist and a questionnaire of attitude towards vaccination were used. The independent variables, were selected based on previous published literature and included: age, sex, education level, income level (Tomans per month, although the Rial is the official currency, Iranians use the Toman in everyday life), amount spent on substance consumption per month, living conditions, drug injection history, prison history, mental illness history, heart disease, infectious diseases, self-reported health status (poor, moderate, good), previous Covid-19 infection (asking if they had ever tested for Covid-19), arrest and detoxification camp during Covid-19 pandemic, ethnicity, marital status, access to COVID-19 vaccination center, current living partner. The attitude towards Covid-19 vaccination was measured by using the 5-point Likert Covid -19 vaccination attitude scale for adults (Co-VASA), ranging from completely disagree (score = 1) to completely agree (score = 5) [38].

Questions in this scale measured understanding of Covid-19 disease transmission, trust in the Covid-19 treatment staff and understanding of the advantages of Covid-19 vaccination. Higher scores suggested a better attitude towards Covid-19 vaccination. The validity and reliability of this scale has been confirmed in previous research [38]. Also, in this study, the reliability was rechecked with Cronbach's alpha and 0.9 was obtained, which indicates the good reliability of the questionnaire.

### Statistical analysis

Descriptive statistics were used as mean and standard deviation (SD) for the quantitative variables, and percentages were used for the qualitative variables. The association between the dependent and independent variables was investigated using chi-square (categorical variables) and Mann–Whitney U tests (continuous variable).

Multivariable logistic regressions with robust standard error estimation via generalized estimating equations were performed to identify factors related to Covid-19 vaccination. Following Hosmer and Lemeshow's "purposeful selection of variables" approach to model building. The dependent variable was Covid-19 vaccine compliance in the model. A *p*-value of < 0.05 was considered statistically significant in the analysis.

Variables with a significance level of ≤ 0.2 were considered in the multivariable model in the univariate analysis, after checking the co-linearity and variance inflation factors between the variables. Also, based on the importance of variables and the possible associations between them (correlation, confounding and interactions), the forward logistic regression model was performed using SPSS software version 20.

## Results

Overall, a total of 386 people who use substances were recruited for the study. The response rate was 91.90%. Among the respondents, 74.1% (*n* = 286, CI 95%, 70.2–79.3) stated that they had received at least two doses of the COVID-19 vaccination. About 1 in 4 (24.9% *n* = 96, CI 95%, 28.8–20.7) participants reported receiving fewer than two doses of Covid-19 vaccine (Table 1).

**Table 1:** Frequency distribution of COVID-19 vaccination among participants

At least two doses of COVID-19 vaccination	Number	percent	Lower	Upper
Yes	286	74.1	70.2	79.3
No	96	24.9	20.7	28.8
No response	4	1		
Total	386	100		

Most (84%) participants were male (*n* = 304) and about one-third (*n* = 132, 35.1%) were aged over 50 years. One-third (33%) reported secondary school education with marital status of single (*n* = 110, 28.8%) and divorced (*n* = 114, 29.8%). See Tables 2 and 3.

**Table 2:** The association between demographic variables and COVID-19 vaccination

Variable		Total samples	COVID 19 Vaccination		*P*-value
			**Yes** **(%)**	**No** **(%)**	
Age	Less than 30	64 (17)	46 (16.3)	18 (19.1)	0.082
	31–50	180 (47.9)	128 (45.5)	52 (55.3)	
	Over 50	132 (35.1)	108 (38.3)	24 (25.5)	
Sex	Male	304 (84)	220 (81.5)	84 (91.3)	0.027
	Female	58 (16)	50 (18.5)	8 (8.7)	
Income level Iranian currency	Less than 2 million	1000 (26.3)	70 (24.6)	30 (31.3)	0.198
	2–5 million	194 (50.1)	154 (54.2)	40 (41.7)	
	Over 5 million	86 (22.6)	60 (21.1)	26 (27.1)	
Living condition during COVID-19 pandemic	Rented house	92 (24.1)	70 (24.5)	22 (22.9)	0.546
	Father’s house	30 (7.9)	22 (7.7)	8 (8.3)	
	Motel	16 (4.2)	14 (4.9)	2 (2.1)	
	Shelter	124 (32.5)	94 (32.9)	30 (31.3)	
	Street /park	86 (22.5)	64 (22.4)	22 (22.9)	
	Personal house	24 (6.3)	14 (4.9)	10 (10.4)	
	Others	10 (2.6)	8 (2.8)	2 (2.1)	
Current living partner	Living alone	140 (36.6	96 (33.6)	44 (45.8)	0.031
	Living with family	84 (22)	62 (21.7)	22 (22.9)	
	Living with friends /colleagues	158 (41.4)	128 (44.8)	30 (31.3)	
Education level	Illiterate	18 (4.7)	14 (4.9)	4 (4.2)	0.013
	Primary school	80 (20.9)	68 (23.8)	12 (12.5)	
	Secondary school	126 (33)	82 (28.7)	44 (45.8)	
	High school diploma	120 (31.4)	90 (31.5)	30 (31.3)	
	University	38 (9.9)	32 (11.2)	6 (6.3)	
Ethnicity	Fars	166 (43.5)	118 (41.3)	48 (50)	0.011
	Others (non-Fars)	216 (56.5)	168 (58.7)	48 (50)	
Marital status	Single	110 (28.8)	72 (25.2)	38 (39.6)	0.034
	Married and with spouse	66 (17.3)	52 (18.2)	14 (14.6)	
	Married and away from spouse	92 (24.1)	7 (26.6)	16 (16.7)	
	Divorced-widow	114 (29.8)	86 (30.1)	28 (29.2)	
Self-reported health status	Poor	78 (20.5)	54 (19)	24 (25)	0.394
	Moderate	162 (42.6)	120 (42.3)	42 (42.3)	
	Good	140 (36.8)	110 (38.7)	30 (38.7)

**Table 3:** The association between variables related to Covid-19 vaccination

Variable		Total samples	Covid-19 Vaccination		*P*-value
			**Yes** **(%)**	**No** **(%)**	
Cost of Substance consumption per month	Less than 2 million	208 (54.1)	156 (55.1)	52 (55.3)	0.801
	2–5 million	74 (19.5)	58 (20.3)	16 (17)	
	Over 5 million	96 (24.5)	70 (24.6)	26 (27.7)	
Lifetime drug injection history	No	304 (83.1)	226 (81.9)	78 (86.7)	0.299
	Yes	62 (16.9)	50 (18.1)	12 (13.3)	
Lifetime prison history	Yes	266 (69.6)	196 (68.5)	70 (72.9)	0.412
	No	116 (30.4)	90 (31.5)	26 (27.1)	
History of sexually transmitted infections	Yes	60 (15.7)	48 (16.8)	12 (12.5)	0.318
	No	324 (84.3)	238 (83.2)	84 (87.5)	
History of mental illness	Yes	94 (25)	64 (22.9)	30 (31.3)	0.152
	No	282 (75)	216 (77.1)	66 (68.8)	
History of chronic diseases	Yes	104 (27.7)	72 (25.7)	32 (33.3)	0.158
	No	272 (72.3)	208 (74.3)	64 (66.7)	
History of COVID-19 infection	Yes	108 (28.4)	82 (28.9)	26 (27.1)	0.733
	No	272 (71.6)	202 (71.1)	70 (72.9)	
Access to vaccination center	High	272 (71.6)	226 (79.6)	46 (47.9)	0.001
	Low	108 (28.4)	58 (20.4)	50 (52.1)	
History of arrest for detox camp during COVID-19 pandemic	Yes	216 (66.3)	164 (67.2)	52 (63.4)	0.522
	No	110 (33.7)	80 (32.8)	30 (36.6)	
Attitude towards COVID-19 vaccination	Mean and SD	56.89(11.23)	59.68 (9.26)	48.11 (12.09)	0.001
Type of common Substance used	Opium	74 (19.5)	62 (21.8)	12 (12.5)	0.031
	Heroin	148 (38.9)	102 (35.9)	46 (47.9)	
	Meth and heroin cocktail	34 (8.9)	26 (9.2)	8 (8.3)	
	Methamphetamine	98 (25.8)	70 (24.6)	28 (29.2)	
	Others including: cannabis, ketamine, LSD, ecstasy	26 (6.8)	24 (8.5)	2 (201)	

The results of the bivariate analysis showed no association between age, income level, cost of substance consumption per month, living condition, history of drug injection, lifetime prison history, history of mental illness, history of heart disease, history of infectious disease, self-reported health status, history of Covid-19 infection, history of arrest for detoxification camp during Covid-19 pandemic and history of Covid-19 vaccination (*p* > 0.05) (See Tables 2 and 3).

A significant association was found between sex, current living partner, level of education, ethnicity, marital status, access to COVID-19 vaccination centers, and attitude toward COVID-19 vaccination with receiving COVID-19 vaccination (*p* < 0.05) (Tables 2 and 3).

The results of Table 4 and multivariable analysis show that the variables of positive attitude towards vaccination, education level, ethnicity, income level, and having access to Covid-19 vaccination centers had a significant association with being vaccinated for Covid-19 (*p* < 0.05) In other words, these variables are predictors of Covid-19 vaccination. Participants with high school diplomas were 1.17 times more likely to report at least two doses of the Covid-19 vaccine than participants who were illiterate (OR of 1.17, CI 95%: 1.03–1.81). Participants in this study with a higher mean score of positive attitudes towards Covid-19 vaccination were also more likely to report having been vaccinated with two Covid-19 doses (OR of 1.12, CI 95%: 1.08–1.17). Individuals of non-Fars ethnicities (OR of 0.33 CI 95%: 0.13–0.81) had a lower chance of receiving Covid-19 vaccination than people of Fars ethnicity. People with a monthly income of 2–5 million Tomans had a higher chance of receiving Covid-19 vaccination than people with a monthly income of fewer than 2 million Tomans (OR of 1.27, CI 95%: 1.09–1.8). People with low access to vaccination centers had a lower chance of receiving Covid-19 vaccination (OR of 0.17, CI 95%: 0.08–0.36). Also, there was no significant association between other variables and Covid-19 vaccination. The results of the Hosmer–Lemeshow test showed that the model had an appropriate goodness of fit with the data (*P* = 0.5).

**Table 4:** Adjusted Association of Variables with Receiving COVID-19 Vaccination Using Multivariable Logistic Regression

Variables		OR	95% confidence interval		*P*-value
			Lower	Upper	
Age	Less than 30	-	-	-	0.149
	31–50	0.36	0.10	1.24	0.107
	Over 50	0.46	0.19	1.08	0.075
Positive attitude towards COVID-19 vaccination		1.12	1.08	1.17	0.001
Educational level	Illiterate	-	-	-	0.047
	Primary school	0.52	0.06	4.18	0.547
	Secondary school	0.64	0.12	3.26	0.592
	High school diploma	1.17	1.03	1.81	0.027
	University	0.29	0.06	1.31	0.108
Ethnicity	Fars	-	-	-	0.038
	non-Fars	0.33	0.13	0.81	0.017
Marital status	Single	-	-	-	0.294
	Married and with spouse	0.77	0.30	1.96	0.582
	Married and away from spouse	1.5	0.50	4.54	0.466
	Divorced-widow	2.2	0.77	6.2	0.138
Income level	Less than 2 million	-	-	-	0.018
	2–5 million	1.27	1.09	1.8	0.019
	Over 5 million	1.29	0.51	3.24	0.582
Sex (male versus female)		0.338	0.12	1.24	0.112
History of mental illness (yes vs no)		0.62	0.27	1.43	0.269
Access to vaccination center (yes vs no)		0.17	0.08	0.36	0.001
History of chronic diseases (yes vs no)		0.64	0.29	1.43	0.281
Current living partner	Living alone	-	-	-	0.411
	Living with family	0.58	0.26	1.30	0.192
	Living with friends/colleagues	0.65	0.22	1.93	0.443
Substance most commonly used last month	Opium	-	-	-	0.066
	Heroin	0.25	0.02	2.31	0.224
	Methamphetamine and heroin	0.47	0.06	3.51	0.464
	Methamphetamine	0.12	0.014	1.16	0.068
	Others	0.16	0.02	1.33	0.091

## Discussion

Our study is among the first to examine the prevalence of Covid-19 vaccination uptake among people using illicit substances in Iran. We found that 74.1% of the participants have received at least two doses Covid-19 vaccine, which is more than the reported vaccination rate in similar studies in the United States (68%) [39] and Spain (71%) [13]. Explanations for the higher vaccination uptake in the present study may be the different timing of this study (three years after the start of the Covid-19 pandemic, and two years after the approval of the Covid-19 vaccine and the global vaccination campaign). The coverage and availability of Covid-19 vaccinations in Iran was also excellent for the general population. This suggests that efforts to reduce doubt and promote vaccination uptake in people using illicit substances has been somewhat effective for those recruited for this cross-sectional study.

Not surprisingly, findings suggest that those people who use substances who have a positive attitude towards Covid-19 vaccination were more likely than those with a negative attitude to have had at least two doses of the vaccine. This is in line with data from the USA where it has also been shown that a positive attitude toward Covid-19 vaccination is correlated to higher rates of vaccination completion [13, 39]. Studies have shown that trust in doctors and the healthcare system increases the willingness to be vaccinated [40]. This suggests that the stigma and discrimination experienced in healthcare centers by the population can also impact vaccine uptake and compliance. Therefore, interventions that are framed around utilizing reliable information sources, including harm reduction activists and trusted peers can increase trust in the health care system and in turn promote vaccination compliance. Previous studies have shown that people who have received direct vaccination advice from healthcare staff were the ones who also had greater rates of vaccination compliance [13, 41].

A systematic review study conducted by Shakeel and colleagues showed that low vaccine acceptance is associated with lower levels of education and awareness [42]. In line with this finding, in the present study, people with a high school diploma were more likely to receive Covid-19 vaccinations when compared to people who were classified as illiterate. Other studies have also shown an association between education and vaccination uptake [43, 44], and also between education and vaccine hesitancy [18, 45].

In the present study, non-Fars participants had a lower chance of receiving vaccination than the Fars ethnic population. People who use substances are highly stigmatized in Iranian society and it may be that the non-Fars population as a minority ethnicity in Tehran, experience this even more. It is possible that people with non-Fars ethnicity are less likely to attend vaccination centers because of their different socio-cultural beliefs and difficulty in communication due to the language. In this regard, studies have shown that ethnic minority and racial groups experience higher rates of Covid-19 infection and death [46, 47], which may be partly due to non-compliance with preventive behaviors. In line with this finding, some studies have shown that African American people including those who use illicit substances are more hesitant and less likely to get vaccinated than the general population [14, 40, 48].

The findings of the present study show that the Covid-19 vaccination rates among low-income participants were less than others in the study. In line with this finding, previous research has shown that people from lower social classes less likely to be vaccinated for other health issues [49]. Previous studies have emphasized structural issues including financial incentives as ways to improve Covid-19 vaccination uptake and completion [50]. These also include providing transportation services, provision of syringes and needles, and food for those who currently inject drugs.

The results of the present study showed that people who had less access to vaccination centers had a lower chance of receiving a vaccination, and this finding is consistent across other studies [13, 51]. Previous studies have shown that efforts to improve vaccination coverage can partially remove the barriers to vaccination [40], so the establishment of clinics and temporary vaccination centers near the places where people inject their Substances can increase the opportunities for vaccination [52].

In the current study, no association was observed between age, marital status, history of chronic diseases, and the chance of receiving the vaccination. Contrary to the results of this study, other studies [39] have observed that younger people have less chance of receiving Covid-19 vaccination [53]. Studies have also shown that PWIDs with comorbidities are more hesitant to receive Covid-19 vaccination [18], and the willingness to receive vaccination in the general married population is higher than for others [54]. Finally, in this study no association was found between the type of substance used and vaccination, which is in line with other studies that have shown hesitation in receiving a vaccine is not correlated to the type of substance used [22, 39].

### Limitations

Despite the strengths of the present study, including conducting a study on people who use substances and appropriate sampling, study design and analysis the results of the present study should be interpreted with some limitations in mind. There was no gold standard to determine vaccination and diagnosis rates beyond self-report which may expose the study to social desirability bias and inaccuracy. The cross-sectional nature of this study, means that no causal association between variables could be determined. Convenience sampling method and considering only the people who use substances in Tehran in this study, limits the generalizability of the findings to the population of people who use substances in Iran more widely.

## Conclusion

Covid-19 vaccine uptake was found to be relatively high among people using drugs in this study. Higher levels of education, Fars ethnicity, higher income levels, having a positive attitude towards vaccination and access to vaccination centers were the most important predictors for people who received at least two doses of the Covid-19 vaccine. Developing such research in other cities in Iran and different diverse populations is proposed to understand more comprehensively the coverage of Covid-19 vaccination. Policymakers should consider the education and income levels of people when designing strategies for increased and better accessibility to the vaccination centers as well as in designing future interventions to improve vaccination rates for among people who use substances across Iran.

## Data Availability

No datasets were generated or analysed during the current study.

## Abbreviations

CI: Confidence interval
Co-VASA: The Covid-19 Vaccination Attitude Scale for Adults
